# Impact of ERCC2 Gene Polymorphisms on OSCC Susceptibility and Clinical Characteristics

**DOI:** 10.1055/s-0041-1722952

**Published:** 2021-02-18

**Authors:** ML Avinash Tejasvi, Gopal Maragathavalli, Putcha Uday Kumar, M. Ramakrishna, Vijaya Raghavan, Anulekha Avinash CK

**Affiliations:** 1 Department of Oral Medicine and Radiology, Kamineni Institute of Dental sciences, Narketpally, Saveetha University, Chennai, India; 2 Department of Oral Medicine and Radiology, Saveetha Dental College and Hospitals, Chennai, India; 3 Department of Pathology & Microbiology, National Institute of Nutrition, Hyderabad, India; 4 Department of Radiation Oncology, MNJ Institute of Oncology & Regional Cancer Centre, Hyderabad, India; 5 Department of Research and Development, Saveetha University, Chennai, India; 6 Department of Prosthodontics, Kamineni Institute of Dental Sciences Narketpally, Telangana, India

**Keywords:** oral cancer, ERCC2, DNA repair, platinum-based chemotherapy

## Abstract

**Background** DNA repair systems play an important role in maintaining the integrity of the human genome. Deficiency in the repair capacity due to either mutations or inherited polymorphisms in DNA repair genes may contribute to variations in the DNA repair capacity and subsequently susceptibility to cancer.

**Objectives** This study aimed to investigate the association between Excision repair cross-complementation groups 2 (ERCC2) single nucleotide polymorphisms (SNPs rs1799793 and rs13181) and the response to platinum-based chemotherapy among patients with oral squamous cell carcinoma (OSCC).

**Methodology** Polymerase chain reaction‐based restriction fragment length polymorphism analysis was used to determine the polymorphism from a total of 150 OSCC patients and 150 normal tissues of same patients were collected as controls for this study.

**Results** ERCC2 GA (Asp312Asn) AC (Lys751Gln) genotypes were significantly associated ( *p =* 0.0001 and *p* = 0.0004, respectively) with OSCC patients, when compared with the controls. These findings suggest that potentially functional SNPs in *ERCC2* may contribute to OSCC risk. This study highlights the genetic variant that might play a role in mediating susceptibility to OSCC in this population. An understanding of DNA repair gene polymorphisms might not only enable risk assessment, but also response to therapy, which target the DNA repair pathway.

## Introduction

Cancer describes a group of diseases characterized by the uncontrolled growth and spread of abnormal cells. Cancer is the result of multiple environmental and genetic risk factors, as well as gene–environment interactions. 1 Among genetic factors, genetic and epigenetic mutations, such as aberrant DNA methylation, can lead to carcinogenesis. 2 The relationship between genetic polymorphisms and the risk of cancer has been widely researched. The nucleotide excision repair (NER) pathway, a highly powerful and sophisticated DNA damage removal pathway, has been believed to play important roles in cancer progression and response to platinum-based chemotherapy. 3 4 Excision repair cross-complementation groups 2 (ERCC2) are gene encoding one of the key enzymes in NER pathway. 5 *ERCC2* gene, also called the xeroderma pigmentosum group D ( *XPD* ) gene, is located at chromosome 19q13.3. It comprises of 23 exons and spans approximately 54,000 base pairs. 6 It is an important component of the transcription factor IIH that is involved in NER of UV-induced damage and removal of bulky adducts. 7

Genetic variations in ERCC2/ *XPD* are associated with defects in the NER mechanism resulting in autosomal recessive DNA repair disorders. 8 Two single nucleotide polymorphisms (SNPs) in *XPD* , p.Asp312Asn (rs1799793) and p.Lys751Gln (rs13181), have been shown to be involved in susceptibility to various types of cancer in addition to various inherited and age-related diseases. 7 9 The rs13181 affects an ATP-binding site of ERCC2 and destroys its helicase activity, which is important for NER, but does not affect its transcriptional activity. 10 The lysine at codon 751 is assumed to be involved in interactions with the substrate of ERCC2, thus any substitution at this residue may produce changes in its function which can impair the DNA repair capacity. 8 Since the association of the *ERCC2* Lys751Gln (rs13181) polymorphism with OSCC risk was first reported in 2002, there are additional investigations of the association between Lys751Gln and risk of ESCC among different ethnicities but the results have been mixed or conflicting, likely due to a relatively small sample size in each of the published studies. 6 7 The purpose of this study was to investigate whether the two SNPs were associated with resistance to cisplatin chemotherapy.

## Materials and Methods

### Study Population

Oral squamous cell carcinoma (OSCC) patients were assessed on the basis of clinical and pathological examinations. This study is a hospital-based split mouth study conducted in South Indian population. All incidents of OSCC cases were newly diagnosed. During the study period Ethics Committee approved the study for the benefit of humans in general. The procedures followed were in accordance with the ethical standards of responsible committee of the Institute/Hospital, to participate in a face-to-face interview using a structured questionnaire.

### Inclusion and Exclusion Criteria

We included all patients with OSCC who were treated with cisplatin-based chemotherapy. Patients with confirmed OSCC who gave their consent were included. All patients who refused to give their consent were excluded.

### Sample for the Study

Based on the above criteria, tissue samples from a total of 150 OSCC patients and normal tissues of same patients were collected as controls for this study. Sampling was done from Cancer Hospital, between the period June 2016 to August 2018. Senior pathologists confirmed all diagnoses. We interviewed and collected the data about the patient's demographic factors. We collected the information on age, smoking, and previous cancer diagnoses. Participants were also asked about their family history of cancer, and the clinical information for these cases was obtained from medical records like tumor size, stage, and whether they were receiving chemotherapy. A total of 150 patients were treated with cisplatin-based chemotherapy.

### Collection of Tissue Samples

Incisional biopsy was done from the representative oral cancer tissue and sent to histopathology laboratory for diagnosis. All the samples were diagnosed mainly as OSCC. Healthy tissue samples were also collected from same patients, which were used as controls.

### Genotyping

**Fig. 1:**
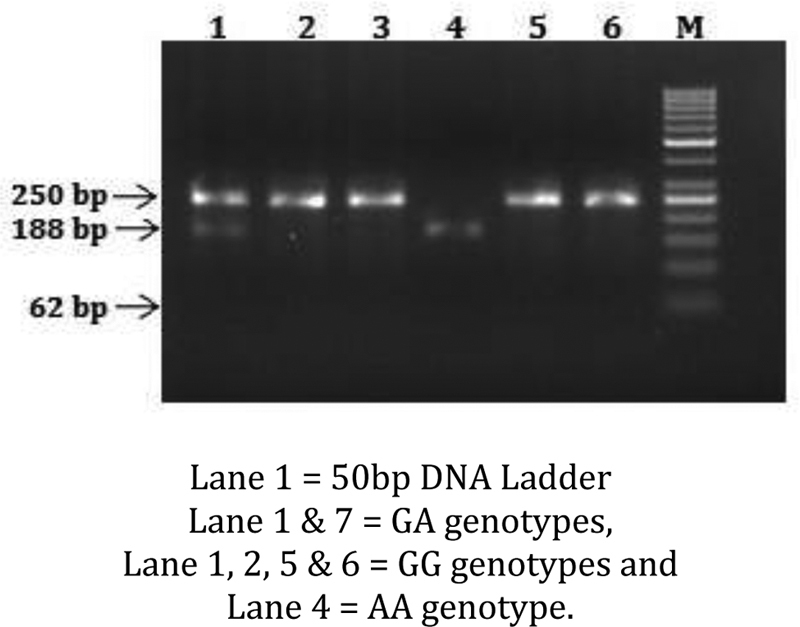
ERCC1 rs1799793, PCR products after restriction digestion with Tai1 on 3% agarose gel. PCR, polymerase chain reaction.

**Fig. 2:**
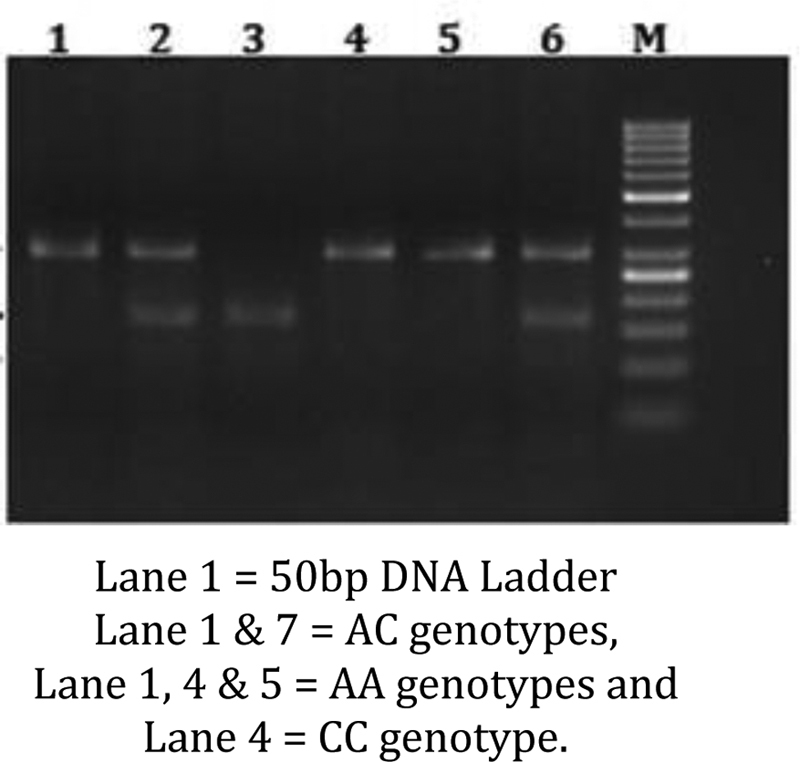
ERCC1 rs rs13181 PCR products after restriction digestion with Mb *o* II on 3% agarose gel. PCR, polymerase chain reaction.

Peripheral blood was collected from the patients prior to their treatment in EDTA-anticoagulant tubes. Genomic DNA was prepared from peripheral blood leucocytes using a QIAamp Blood Mini Kit (Blossom, Taipei, Taiwan) and genotyping assays for the XPD polymorphisms (rs1799793, Asp312Asn; rs13181, Lys751Gln of ERCC2/XPD) according to our previous paper (6). Briefly, the following primers were used for XPD Asp312Asn: 5′-TGGCCCCTGTCTGACTTGTCCC-3′ and 5′- GACGGGGAGGCGGGAAAGGGACT-3′; for XPD Lys751Gln: 5′-ACTTCATAAGACCTTCTAGC and 5′-GATTATACGGACATCTCCAA-3′. The following cycling conditions were performed: one cycle at 94°C for 5 minutes; 35 cycles of 94°C for 30 seconds, 55°C for 30 seconds, and 72°C for 30 seconds; and a final extension at 72°C for 10 minutes. The PCR products were studied after digestion with Hpy99I, EarI, and Bme1580I, restriction enzymes for XPD Asp312Asn (cut from 250 bp A type into 188 + 62 bp G type), and Lys751Gln (cut from 326 bp C type) into 127 + 199 bp A type; ( Figs. 1 and 2 ).

### Statistical Analysis

The demographic and clinical data were expressed as number ( *N* ) and percentage (%). Statistical data analysis was done using Medcalc. Statistical significance was set as *p* < 0.05. Values were expressed as percentage and mean. Data were compiled according to the genotype and allele frequencies.

## Results

### Biological Characteristics

The distribution patient's biological characteristics and selected risk factors are shown in Table 1 . Age range for OSCC patients was 9 to 87 years in males and 27 to 75 years in females and in controls 21 to 80 years in males and 22 to 87 years in females. However, many of the ages mentioned in case sheets or given by patients were arbitrary, exact age of 150 OSCC patients (males 81 and females 69) and 150 controls (males 91 and females 59) was available, hence analysis was performed with those, mean age at which OSCC was identified as 9–87/49.30 ± 15.55 in males and 27–75/84.20 ± 11.26 in females years. To understand the role of gene mutations/polymorphisms in onset of the disease, the patients were divided into four categories, <25 years (1.33%), 26 to 45 years (32.00%), 46 to 65 years (54.00%), and above 66 years (20.12%). Highest percentage of OSCC patients was identified between 46 and 65 years. Regarding the primary tumor site, there was a neat predominance on the BM adding up to 56 patients (37.33%), followed by tongue adding up to 33 patients (22.0%), then mandible, oral cavity, and RMT adding up to 12, 10, and 7%. In the present study, high percentage was identified in BM patients and low percentage was observed in BOT, FOM maxilla, palate, and lip sites. The stage of a cancer is a descriptor (usually numbers I to IV) of how much the cancer has spread. The stage often takes into account the size of a tumor. In the present study stage III showed the highest frequency (40%) when compared with stage II (22%) and stage IV (31.33%), and other types of tumor grades like stage I (6.67%) showed very low frequency when compared with other staging groups. The percentage of patients with family history was 5% with OSCC. In the present study, majority of the patients, 40.7%, had received radiation therapy with adjuvant chemotherapy, 23.3% underwent surgery, followed by surgery with adjuvant chemotherapy 14.0%. A total of 13.3 and 0.7% of the patients received radiation and chemotherapy alone. Surgical excision and/or adequate radiation therapy remain the most effective means of treating the patients with OSCC.

**Table 1:** Demographic and clinicopathological characteristics among healthy cancer free controls and patients with OSCC

Clinical characteristics	*N* = 150
Gender	
Males	81 (54%)
Females	69 (46%)
Range mean (Males)	9–87/ 49.30 ± 15.55
Range mean (Females)	27–75/84.20 ± 11.26
Age distribution	
<45	50 (33%)
46–65	81 (54%)
>65	19 (13%)
Tumor site	
Buccal mucosa	56 (37.33%)
Tongue	33 (22%)
Ventral surface of tongue	2 (1.3%)
Floor of the mouth	6 (4%)
Lip	4 (2.67%)
Alveolar mucosa of mandible	18 (12%)
Alveolar mucosa maxilla	3 (2%)
Palate	3 (2%)
Retro molar trigone	11 (7.33%)
Oral vestibular sulcus	14 (9.3%)
Clinical stages	
Stage 0–II	43 (28%)
Stage III–IV	107 (72%)
Habitual risk	
Alcoholics	2 (1.3%)
Smokers	12 (8%)
Chewing	46 (30.6%)
Combination risk factors	
Alcohol + smoking	19 (12.6%)
Alcohol + chewing	35 (23.33%)
Smoking + chewing	4 (2.6%)
Alcohol + smoking + chewing	15 (10%)
No habits	17 (11.33%)

### ERCC2 Asp312Asn (rs 1799793) Genotype Distribution in OSCC Patients and Control Subjects

ERCC2 Asp312Asn (rs 1799793) polymorphism was analyzed by polymerase chain reaction-restriction fragment length polymorphism (PCR-RFLP) and the PCR product (250bp) was digested with Hpy99I restriction enzyme. The DNA fragments were then separated using 2% agarose gel and detected by ethidium bromide staining. OSCCC patients showed 57% GG, 41% GA, and 25% AA genotypes when compared with controls that had 84% GG, 15% GA, and 1% AA genotypes. Table 2 shows results for the Asp312Asn (rs 1799793) polymorphism; there was a significant difference in the distribution of GA and GA + AA genotypes between patients and controls. Both the heterozygous (GA) and hetro and homozygous (AA) genotypes were more in patients when compared with controls; however, only the GA genotype was significantly associated with OSCC (OR0.74, 95% CI 0.39–1.41, *p* -value = 0.0001). GA + AA genotype was also significantly associated with OSCC (OR 3.90, CI 2.26–6.72, and *p* -value = 0.0001).

**Table 2:** Distribution of ERCC2 (rs 1799793) polymorphism genotypes in OSCC patients and controls

ERCC2 SNP rs 1799793	Case group *n* = 150	Control group *n * = 150	Odds ratio	95% CI	*p* -Value
GG	86 (57%)	126 (84%)	0.74	0.39–1.41	0.36
GA	60 (41%)	22 (15%)	3.87	2.22–6.77	0.0001
AA	4 (25)	2 (1%)	2.02	0.36–11.2	0.41
GA + AA	64 (43%)	24 (16%)	3.90	2.26–6.72	0.0001

Abbreviations: 95% CI, 95% confidential intervals; OR, odds ratio.

*p* = < 0.05 (significant).

### ERCC2 Lys751Gln (rs13181) Genotype Distribution in OSCC Patients and Control Subjects

ERCC2 Lys751Gln (rs13181) polymorphism was analyzed by PCR-RFLP and the PCR product (326bp) was digested with EarI restriction enzyme. The DNA fragments were then separated using 2% agarose gel and detected by ethidium bromide staining. OSCCC patients showed 71% AA, 25% AC, and 4% CC genotypes when compared with controls that had 90% GG, 9% AC, 1% CC genotypes. Table 3 shows results for the Asp312Asn (rs 1799793) polymorphism; there was a significant difference in the distribution of AC and AC + CC genotypes between patients and controls. Both the heterozygous (AC) and hetro and homozygous (CC) genotypes were more in patients when compared with controls, however, only the AC genotype was significantly associated with OSCC (OR 3.29, 95% CI 1.70–6.38, *p* -value = 0.0004]. AC + CC genotype is also significantly associated with OSCC (OR 3.73, CI 1.97–7.07, and *p* -value = 0.0001).

**Table 3:** Distribution of ERCC2 (rs13181) polymorphism genotypes in OSCC patients and controls

ERCC2 SNP rs13181	Patients *n* = 150	Controls *n* = 150	Odds ratio	95% CI	*p* -Value
AA	106 (71%)	135 (90%)	0.26	0.14–0.50	0.0001
AC	38 (25%)	14 (9%)	3.29	1.70–6.38	0.0004
CC	6 (4%)	1 (1%)	6.20	0.73–52.2	0.09
AC + CC	44 (29%)	15 (10%)	3.73	1.97–7.07	0.0001

Abbreviations: 95% CI, 95% confidential intervals; OR, odds ratio; OSCC, oral squamous cell carcinoma.

*p* ≤ 0.05 (significant).

### Correlation ERCC2 Asp312Asn (rs 1799793) and Lys751Gln (rs13181) Genotypes with OSCC Clinical Characteristics of OSCC Patients

ERCC2 Asp312Asn (rs 1799793) and Lys751Gln (rs13181) genotypes were correlated with demographic factors like gender, age, histology, staging, and habitual risks of OSCC patients to see the effect of genetic polymorphism in modulating the risk of developing OSCC in association with all demographic factors. Comparison of clinicopathologic characteristics and ERCC2 Asp312Asn (rs 1799793) and Lys751Gln (rs13181) *ERCC2* genotypes revealed no significant differences ( Tables 4 and 5 ).

**Table 4:** Correlations of clinical characteristics of oral squamous cell carcinoma patients with *ERCC2* Asp312Asn (rs 1799793) polymorphism

Clinical characteristics	Cases *N* = 150 (%)	Wild type GG *N * = 86 (%)	Heterozygous GA *N* = 60 (%)	Homo mutant AA *N* = 4 (%)
Age distribution				
<45	50 (33%)	31 (62%)	18 (36%)	1 (2%)
46–65	81 (54%)	43 (53%)	37 (46%)	1 (1%)
>65	19 (13%)	12 (63%)	5 (26%)	2 (11%)
Tumor site				
Buccal mucosa	56 (37%)	29 (68%)	26 (29%)	1 (3%)
Tongue	33 (22%)	17 (52%)	15 (45%)	1 (3%)
Bottom of the tongue	2 (1%)	2 (100%)	0 (0%)	0 (0%)
FOM	6 (4%)	5 (50%)	1 (50%)	0 (0%)
LIPS	4 (3%)	4 (100%)	0 (0%)	0 (0%)
Mandible	18 (12%)	10 (44%)	7 (50%)	1 (6%)
Maxilla	3 (2%)	3 (33%)	0 (67%)	0 (0%)
Palate	3 (2%)	3 (67%)	0 (33%)	0 (0%)
Retromolar Trigon	11 (8%)	7 (55%)	4 (45%)	0 (0%)
Oral cavity	14 (9%)	6 (64%)	7 (36%)	1 (0%)
Stages				
Stage 0–II	43 (29%)	26 (60%)	15 (35%)	2 (5%)
Stage III–IV	107 (71%)	60 (56%)	45 (42%)	2 (2%)
Habitual risk				
Alcoholics	2 (1%)	1 (50%)	1 (50%)	0 (0%)
Smokers	12 (8%)	7 (58%)	3 (25%)	2 (17%)
Chewing	46 (31%)	25 (54%)	21 (46%)	0 (0%)
Combination risk factors				
Alcohol + smoking	19 (13%)	11 (58%)	8 (42%)	0 (0%)
Alcohol + chewing	35 (23%)	20 (57%)	14 (40%)	1 (3%)
Smoking + chewing	4 (3%)	3 (75%)	1 (25%)	0 (0%)
Alcohol + smoking + chewing	15 (10%)	8 (53%)	7 (47%)	0 (0%)
No habits	17 (11%)	11 (65%)	5 (29%)	1 (6%)

**Table 5:** Correlations of clinical characteristics of oral squamous cell carcinoma patients with *ERCC2* Lys751Gln (rs13181) polymorphism

Clinical characteristics	Cases *N* = 150 (%)	Wild type AA *N * = 106 (%)	Heterozygous AC *N * = 38 (%)	Homo mutant CC *N * = 6 (%)
Age distribution				
<45	50	33 (66%)	15 (30%)	2 (4%)
46–65	81	61 (75%)	19 (24%)	1 (1%)
>65	19	12 (63%)	4 (21%)	3 (16%)
Tumor site				
BM	56	42 (75%)	13 (23%)	1 (2%)
Tongue	33	22 (67%)	10 (30%)	1 (3%)
BOT	2	2 (100%)	0 (0%)	0 (0%)
FOM	6	5 (83%)	1 (17%)	0 (0%)
LIP	4	4 (50%)	0 (50%)	0 (0%)
Mandible	18	13 (72%)	4 (22%)	1 (6%)
Maxilla	3	2 (34%)	0 (0%)	1 (33%)
Palate	3	2 (100%)	1 (0%)	0 (0%)
RMT	11	10 (55%)	1 (27%)	0 (18%)
Oral cavity	14	11 (79%)	3 (21%)	0 (0%)
Stages				
Stage 0–II	43	32 (74%)	9 (21%)	2 (5%)
Stage III–IV	107	74 (69%)	29 (27%)	4 (4%)
Habitual risk				
Alcoholics	2	1 (50%)	1 (50%)	0 (0%)
Smokers	12	7 (58%)	4 (33%)	1 (9%)
Chewing	46	34 (74%)	11 (24%)	1 (2%)
Combination risk factors				
Alcohol + smoking	19	14 (74%)	5 (26%)	0 (0%)
Alcohol + chewing	35	25 (71%)	9 (26%)	1 (3%)
Smoking + chewing	4	2 (50%)	1 (25%)	1 (25%)
Alcohol + smoking + chewing	15	10 (67%)	4 (27%)	1 (6%)
No habits	17	13 (76%)	3 (18%)	1 (6%)

## Discussion

The *ERCC2* gene is located on chromosome 19q13.3, comprises of 23 exons and encodes 760 amino acids. Acting as a single-strand DNA-dependent ATPase, and also a 5′-3′ DNA helicase, the ERCC2 protein participates in both DNA unwinding during NER and transcription initiation by binding to the transcription factor IIH via p44. 8 10 Mutations in *ERCC2* could result in transcription defects and abnormal apoptosis by reducing the BTF2/TFIIH activity, thus leading to a severe but variable depression of NER. 11 ERCC2 also has a second function that is involved in base excision repair of oxidative base damage of the transcribed strand of transcriptionally active gene. Genetic variations in DNA repair genes, which are important in maintaining DNA stability, may affect DNA repair capacity and consequently increase an individual's susceptibility to cancer. Several proteins are involved in DNA repair pathways. Genetic polymorphisms in *ERCC2* gene have been shown to be involved in various malignancies. *ERCC2* genes participate in DNA repair and therefore, when mutated, may contribute to genome instability. SNP at amino acid 751 of *ERCC2* may play an important role in *ERCC2* protein activity. 12 The *ERCC2* 751 polymorphism (rs13181) was associated with higher levels of chromatic aberrations and DNA adducts levels. 13 14 It was reported that *ERCC2* 751(rs13181) AC/CC genotypes were significantly defective in NER and had a modulating effect on DRC. 15 16 These results suggested that *ERCC2* 751 polymorphism (rs13181) could result in a defect in NER and deficient DRC that may be responsible for increased susceptibility of oral cancer. Thus, genetic variants in these genes may be associated with the susceptibility of various types of cancers.

The two SNPs analyzed in the present study were the common SNPs in exons of *ERCC2* gene. SNP rs1799793 is G > A substitution at *ERCC2* codon 312 (exon 10, Asp > Asn) and rs13181 is A > C substitution at *ERCC2* codon 751 (exon 23, Lys > Gln). Numerous studies have explored the relationship between DNA repair gene *ERCC2* polymorphisms and cancer risk. Amit Kumar Mittal reported that the polymorphism rs13181 might be a risk factor for predisposition toward SCCHN and breast cancer among north Indian subpopulations. Bau et al 17 observed a significant difference in the frequency of the *ERCC2* -rs1799793 genotype between the prostate cancer and control groups in Asian populations. Our study observed statistically significant association of ERCC2 polymorphisms with OSCC. The risk of oral cancer might be modified by many other DNA repaired genes in addition to *ERCC2* gene. Further studies with large sample size are greatly needed to verify the results of our study.

Many studies have investigated the potential predictive and prognostic role of ERCC2 (A751C and G312A) genetic polymorphisms on either tumors' response to cisplatin chemotherapy or clinical outcomes. Li et al 2018 18 reported that ERCC2 rs1799793 polymorphism might be a predictor of prognosis in gastric cancer patients subjected to platinum-based chemotherapy. Vella et al 19 has suggested that low ERCC2 expression is associated with increased chemotherapeutic sensitivity and thus considered a predictive marker for patients with ovarian cancer receiving combination of gemcitabine and cisplatin chemotherapy. Smolarz et al 20 ERCC2-Lys751Gln, polymorphisms have been shown to have functional significance and may be in part responsible for the interindividual difference in capacity of DNA repair in the general population and for low DNA repair efficacy in patients with various cancers. Of these studies, many have shown significant associations with these variants.

## Conclusion

In our study, we observed that ERCC2 polymorphisms were significantly associated with OSCC and genotypes revealed no significant differences with clinical parameters. These polymorphisms may also be associated with the clinical sensitivity of platinum-based chemotherapy and could be a potential predictive oral cancer patient.
